# AKT in Bone Metastasis of Solid Tumors: A Comprehensive Review

**DOI:** 10.3390/cancers13102287

**Published:** 2021-05-11

**Authors:** Nico Hinz, Manfred Jücker

**Affiliations:** Center for Experimental Medicine, Institute of Biochemistry and Signal Transduction, University Medical Center Hamburg-Eppendorf, 20246 Hamburg, Germany; hinz_nico@gmx.de

**Keywords:** AKT, protein kinase B, bone metastasis, cancer metastasis, vicious cycle, osteolysis, breast cancer, prostate cancer, solid tumors

## Abstract

**Simple Summary:**

Bone metastasis is a frequent complication of solid tumors and leads to a reduced overall survival. Although much progress has been made in the field of tumor therapy in the last years, bone metastasis depicts a stage of the disease with a lack of appropriate therapeutical options. Hence, this review aims to present the role of AKT in bone metastasis of solid tumors to place the spotlight on AKT as a possible therapeutical approach for patients with bone metastases. Furthermore, we intended to discuss postulated underlying molecular mechanisms of the bone metastasis-promoting effect of AKT, especially in highly bone-metastatic breast, prostate, and lung cancer. To conclude, this review identified the AKT kinase as a potential therapeutical target in bone metastasis and revealed remaining questions, which need to be addressed in further research projects.

**Abstract:**

Solid tumors, such as breast cancer and prostate cancer, often form bone metastases in the course of the disease. Patients with bone metastases frequently develop complications, such as pathological fractures or hypercalcemia and exhibit a reduced life expectancy. Thus, it is of vital importance to improve the treatment of bone metastases. A possible approach is to target signaling pathways, such as the PI3K/AKT pathway, which is frequently dysregulated in solid tumors. Therefore, we sought to review the role of the serine/threonine kinase AKT in bone metastasis. In general, activation of AKT signaling was shown to be associated with the formation of bone metastases from solid tumors. More precisely, AKT gets activated in tumor cells by a plethora of bone-derived growth factors and cytokines. Subsequently, AKT promotes the bone-metastatic capacities of tumor cells through distinct signaling pathways and secretion of bone cell-stimulating factors. Within the crosstalk between tumor and bone cells, also known as the vicious cycle, the stimulation of osteoblasts and osteoclasts also causes activation of AKT in these cells. As a consequence, bone metastasis is reduced after experimental inhibition of AKT. In summary, AKT signaling could be a promising therapeutical approach for patients with bone metastases of solid tumors.

## 1. Introduction

Bone is a common site of metastasis of various cancer entities. More precisely, breast cancer (incidence of bone metastases: ~65–75%), prostate cancer (~65–80%), lung cancer (~30–40%), renal cell cancer (~20–25%), malignant melanoma (~14–45%), bladder cancer (~40%), and thyroid cancer (~60%) preferably metastasize to bone [1,2,3].

The preferential spread of these tumor entities to bone is due to an organ tropism in the multistep process of cancer metastasis [4]. Tumor cells from the primary solid tumor invade into the surrounding stroma via an epithelial-to-mesenchymal transition (EMT) and intravasate into the blood circulation as circulating tumor cells (CTCs) [5]. Distinct venous drainage paths and fenestration of capillaries affect the location of distant metastasis on a mechanical level [6]. Additionally, organ-specific mediators, such as bone-derived growth factors, can act as chemoattractants and facilitate the homing and migration of CTCs with the corresponding receptors through the endothelium of bone [6,7,8]. Furthermore, surface molecules on the bone vascular endothelium serve as a molecular ZIP code, which is compatible with specific adhesion molecules, such as integrins, on the tumor cells, and further support an organ tropism [9]. Therefore, extravasation and colonization to a specific organ, such as bone, require certain properties of cancer cells, which enable them to preferentially metastasize to the bone as an expression of the seed-and-soil theory [8,10]. These properties involve genetic, epigenetic, and signaling alterations and depict a selection advantage of these tumor cells within the bone microenvironment by facilitating, for example, homing, invasion, and the crosstalk of tumor cells with bone cells [4,11,12]. Kang et al. revealed an expression profile of breast cancer cells that preferentially colonize the bone marrow, including the connective tissue growth factor (CTGF), interleukin-11 (IL-11), C-X-C chemokine receptor type 4 (CXCR4), and osteopontin (OPN) [13]. For instance, the chemokine receptor CXCR4 on breast or prostate cancer cells mediates the chemoattractive effect of osteoblast-derived C-X-C motif chemokine 12 (CXCL12) for the homing of tumor cells to the bone marrow [6,14]. 

Once extravasated to the bone, the metastatic cancer cells induce either osteolytic lesions via stimulation of bone-resorbing osteoclasts or osteoblastic lesions through stimulation of bone-forming osteoblasts, which are two extremes of a continuum [15]. Accordingly, metastases from prostate cancer cause mostly osteoblastic lesions but can also contain an osteolytic fraction [16,17]. In contrast, breast cancer and lung cancer form mostly osteolytic lesions with partly osteoblastic elements [18,19]. Tumor-induced osteolysis and bone formation, as well as outgrowth of metastases, take place within an interaction between the metastatic tumor cells and the bone microenvironment, including osteoclasts and osteoblasts. This reciprocal communication between the metastases and bone is also known as the vicious cycle of bone metastasis [18,20]. Within the framework of this model, bone-metastatic tumor cells secrete factors, such as the receptor activator of nuclear factor kappa-B ligand (RANKL), the parathyroid hormone-related protein (PTHrP), or CTGF, which stimulate osteoblasts and osteoclasts. Stimulated bone cells, in turn, secrete factors, such as RANKL and CTGF, or release factors, such as the transforming growth factor β (TGFβ) and the insulin-like growth factor 1 (IGF-1), during osteoclast-mediated bone resorption. These factors, in turn, are able to stimulate the tumor cells [9,15,18,21,22]. This crosstalk between the tumor cells and the bone cells leads to the outgrowth of overt bone metastases [4]. As an example, PTHrP is secreted by breast cancer cells after stimulation with bone matrix-derived TGFβ and indirectly stimulates osteoclasts by promoting RANKL secretion from osteoblasts. Analog to its physiological function, RANKL binds to its receptor RANK on osteoclasts and causes the differentiation and activation of osteoclasts via the nuclear factor kappa-light-chain-enhancer of activated B cells (NF_K_B) signaling. As a consequence, inhibition of the TGFβ/PTHrP/RANKL axis leads to a reduced formation of osteolytic metastases [20,23].

Bone metastasis depicts a stage of cancer, which compromises life quality and life expectancy. Furthermore, the formation of bone metastases causes complications, such as pathological fractures, hypercalcemia, bone pain, and spinal cord compression [3,8,15]. The occurrence of pathological fractures in patients with bone metastases is, in turn, associated with an increased risk of death [24]. Once skeletal metastases have developed, a supportive treatment, for example, with anti-resorptive bisphosphonates, the anti-RANKL monoclonal antibody denosumab, chemotherapy, radioisotopes, radiation therapy, and/or surgery, remains the only therapeutical option in this incurable stage of the disease [3,6,8]. Hence, it is of paramount interest to get further insights into the molecular mechanisms of bone metastasis to identify possible targets for treatment, such as dysregulated signaling pathways. The phosphoinositide 3-kinase (PI3K)/AKT signaling pathway is one of these altered pathways, which are associated with bone metastasis of several cancer entities.

The serine/threonine kinase AKT, or otherwise called protein kinase B, is located downstream of PI3K and gets activated after phosphatidylinositol 4,5-bisphosphate (PIP_2_) is converted to phosphatidylinositol 3,4,5-trisphosphate (PIP_3_) by PI3K [25,26]. More precisely, AKT binds with its pleckstrin homology domain to PIP_3_ [27] and, thus, gets activated through phosphorylation by the phosphoinositide-dependent kinase-1 (PDK1) on threonine 308 [28] and by the mammalian target of rapamycin complex 2 (mTORC2) or other kinases, such as the DNA-dependent protein kinase (DNA-PK) on serine 473 [26,29]. The activity of AKT is negatively regulated by the SH2 domain-containing inositol-5-phosphatase (SHIP) or by the phosphatase and tensin homolog (PTEN) through dephosphorylation of PIP_3_ [30,31]. After activation, AKT dissociates from the cell membrane to phosphorylate and to regulate its substrates in the cytoplasm or nucleus [32]. The more than 100 substrates of AKT are involved in numerous cellular processes, including cell proliferation, survival, protein synthesis, metabolism, migration, and much more hallmarks of cancer [33]. As a consequence, several cancer entities, such as lung cancer, prostate cancer, and breast cancer, show a dysregulated PI3K/AKT signaling. Studies about the role of AKT in these cancer entities revealed a promoting function of the PI3K/AKT signaling in tumorigenicity and tumor progression [34,35,36]. For instance, phosphorylation of AKT on serine 473 predicts a poor clinical outcome in prostate cancer patients [37]. Thus, inhibition of the PI3K/AKT axis showed antitumor effects in prostate cancer, lung cancer, as well as breast cancer cells, and AKT inhibitors, such as MK2206, Perifosine, or Ipatasertib, are widely tested in clinical trials [34,38,39,40].

Additionally, AKT plays a crucial role in bone cells such as osteoclasts and osteoblasts and, therefore, regulates the processes of bone remodeling. As a consequence, AKT1 knockout mice showed an osteopenia phenotype due to a dysfunction of osteoblasts and osteoclasts [41]. In detail, AKT1 and AKT2 are involved in osteoclast differentiation and survival as a part of the RANKL/NF_K_B axis [42,43]. Mechanistically, Moon et al. revealed that an AKT/glycogen synthase kinase 3β (GSK3β)/nuclear factor of activated T-cells c1 (NFATc1) axis is involved in the RANKL-mediated osteoclastogenesis [44]. Furthermore, AKT was shown to mediate the TGFβ1-dependent early phase of osteoblast differentiation [45].

Besides the well-known role of AKT in cancer progression, migration, invasion, and metastasis in general, we sought to review the specific role of AKT in bone metastasis of solid tumors and its contribution to the crosstalk between tumor and bone cells. Thereby, we focused on underlying molecular mechanisms of AKT in bone metastasis of breast, prostate, and lung cancer but also shed light on further tumor entities. In addition, we discussed reports about the direct or indirect inhibition of the AKT signaling in bone metastatic diseases. For this purpose, we performed a systematic literature research using the PubMed/MEDLINE library. The search term “AKT bone metastasis” was used, and relevant synonyms such as “bone metastases” or “protein kinase B” were included. The 496 publications initially found were screened for the following inclusion/exclusion criteria within the title and the abstract:

- Inclusion criteria:
  o Experimental studies, clinical-experimental studies, or reviews dealing with AKT in the process of bone metastasis of solid tumor entities.
  o Experimental studies, clinical-experimental studies, or reviews, which cover drugs or substances used in the treatment of bone metastases of solid tumors and affecting the AKT signaling.
- Exclusion criteria:
  o Publications dealing with metastasis of primary bone tumors, such as osteosarcoma, or dealing with bone metastasis or progression of non-solid tumors, such as multiple myeloma.
  o Studies reporting a role of AKT in metastasis to other organs than bone, or in metastasis in general without an organotropism for bone.
  o Studies, which are not available in the English language.

We finally identified 123 publications dealing with AKT in bone metastasis of one or multiple solid tumors (breast cancer: 52 publications; prostate cancer: 50 publications; lung cancer: 10 publications; malignant melanoma: 3 publications; renal cell carcinoma: 3 publications; bladder cancer: 1 publication; hepatocellular carcinoma: 2 publications; colorectal cancer: 1 publication; gastric cancer: 2 publications; oral squamous cell carcinoma: 1 publication; thyroid cancer: 1 publication). 

With this review, we aim to provide information about AKT or AKT signaling-associated proteins as possible targets for the treatment of bone metastasis. This knowledge could place the spotlight on AKT inhibition to improve the treatment of patients with bone metastases.

## 2. Breast Cancer

Breast cancer has one of the highest rates of bone metastasis among all cancer entities [46]. In regard to the breast cancer subtypes, the hormone receptor-positive breast cancer subtype forms more likely bone metastases than human epidermal growth factor receptor 2 (HER2)-positive or triple-negative breast cancer subtypes [47]. Although breast cancer bone metastases predominantly display osteolytic lesions, they also possess osteoblastic elements. Bone metastases of breast cancer cause severe skeletal-related events, such as bone pain and pathological fractures and, therefore, compromise the survival rate of patients [15].

Activation of AKT was shown to be high in breast cancer cells colonizing the bone marrow or when co-cultured with bone marrow stromal cells. Moreover, the inhibition of AKT by application of MK2206 decreases the proliferation of breast cancer cells in this co-culture in vitro [48]. Additionally, osteocyte-derived conditioned medium and the most abundant protein in bone, collagen I, increases the proliferation of breast cancer cells accompanied by elevated pAKT levels [49]. Khotskaya et al. confirmed these findings by reporting a hyperactive AKT/mammalian target of rapamycin (mTOR)/ribosomal protein S6 kinase beta-1 (S6K1) axis in bone-metastatic breast cancer cells, which contributes to the invasiveness of these cells [50]. Microarray gene expression analysis of human breast cancer probes revealed that, among other pathways, the PI3K/AKT signaling is associated with bone metastasis [51]. In contrast, an analysis of somatic mutations in human metastatic breast cancer probes did not show a difference in AKT or PIK3CA mutations between bone metastases and non-bone metastases [52]. Moreover, Li et al. revealed a downregulated PI3K/AKT signaling in the bone-metastatic tissue of primary breast cancer, whereas skin metastatic tissue shows an upregulated PI3K/AKT signaling [53]. 

**Figure 1:**
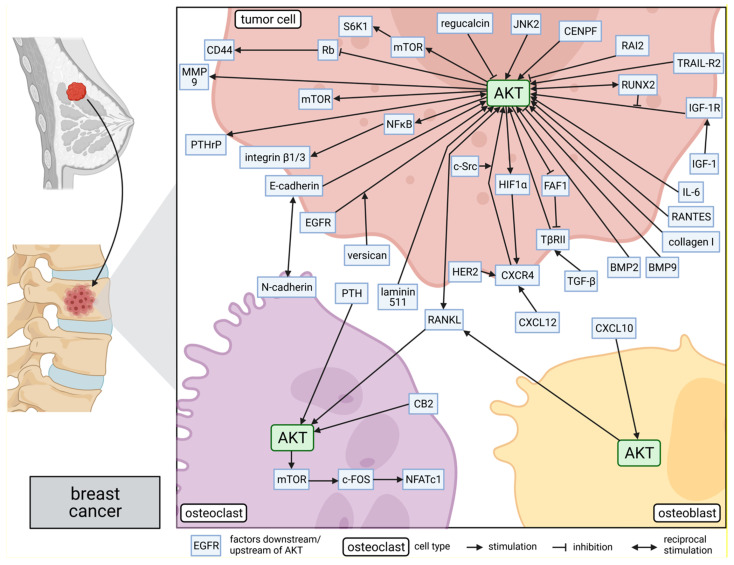
Role of AKT in molecular mechanisms of breast cancer bone metastasis: The side panel on the left side shows schematically the process of breast cancer metastasis to the bone. AKT is involved in several bone metastasis-promoting signaling pathways within breast tumor cells, osteoclasts, and osteoblasts, as shown in the main part of the figure on the right. The position of the proteins indicates their subcellular location in the corresponding cells: intracellular, within the membrane, or secreted. Lines connecting the proteins represent either a promoting or suppressing interaction. This figure was created with BioRender.com.

Nevertheless, the predominant opinion in the literature presents AKT as a promotor of bone metastasis in breast cancer. Several studies shed light on the molecular mechanisms behind this promoting role of AKT for bone metastasis of breast cancer. These mechanisms are discussed in the following and are summarized in Figure 1.

Breast cancer cells preferentially colonize the osteogenic niche during the early stages of bone metastasis and, thus, form bone micrometastases. Mechanistically, E-cadherin-expressing breast cancer cells build heterotypic adherens junctions by binding to N-cadherin on osteogenic cells. These E-N-heterotypic adherens junctions promote the activation of the AKT/mTOR pathway and, thus, leads to the formation of macrometastases [54]. Werner et al. observed that downregulation of the tumor suppressor retinoic acid induced 2 (RAI2) is associated with an early hematogenous dissemination of breast cancer cells to the bone marrow and with an AKT activation in these cells [55].

The expression of centromere protein F (CENPF) as a marker of cell proliferation was revealed to be higher in bone metastases of breast cancer compared to the primary tumor or metastases to other organs. CENPF was further shown to activate the PI3K/AKT/mTORC1 axis to upregulate the expression of PTHrP in breast cancer cells. PTHrP stimulates osteoclastogenesis via upregulation of RANKL secretion in osteoblasts, subsequent binding of RANKL to its receptor RANK on osteoclasts, and activation of the NF_K_B signaling [56]. PTHrP was further shown to activate AKT in breast cancer cells and, thus, potentially promotes proliferation, migration, and invasion [57]. 

In 81.8% of primary HER2-positive breast cancer with bone metastases, pAKT levels were elevated, and the HER2/CXCR4/AKT pathway was suggested to play an important role in bone metastasis [58]. As stated above, the CXCL12/CXCR4 axis plays a pivotal role in homing and invasion of bone-metastatic breast cancer cells [19]. Ablation of CXCR4 in breast cancer cells was further shown to inhibit bone metastasis in a mouse model by attenuating the PI3K/AKT/matrix metallopeptidase 9 (MMP9) axis. MMP9, in turn, promotes invasion and, therefore, metastasis of cancer cells [59]. The bone metastasis-promoting function of the CXCL12/CXCR4 axis is at least in part mediated by an activation of AKT [60,61]. Furthermore, bone metastases are more frequently formed by breast cancer cells with an expression of the hypoxia-inducible factor 1α (HIF-1α), a transcription factor responding to hypoxic conditions. HIF-1α, in turn, is stabilized by AKT and was shown to cause an upregulation of CXCR4 [62]. Moreover, the activity of the proto-oncogene tyrosine-protein kinase Src (c-Src) is associated with late-onset bone metastasis in human breast cancer and is associated with intraosseous breast tumor outgrowth in mice. This effect is mediated by a c-Src-dependent enhanced AKT response to CXCL12 [63]. Furthermore, cancer-associated fibroblasts drive the selection of bone-metastatic breast cancer cells by selecting highly CXCL12- and IGF-1-responsive cells with an Src-dependent sensitivity for CXCL12- and IGF-1-mediated AKT activation [64]. Interestingly, Fritsche et al. revealed that a knockdown of the pro-apoptotic TNF-related apoptosis-inducing ligand receptor 2 (TRAIL-R2) reduces pAKT and CXCR4 levels in breast cancer cells and leads to reduced bone metastasis in vivo [65]. 

Several factors of the bone microenvironment, such as the growth factors TGFβ or IGF-1, were shown to play a crucial role in the activation of AKT in bone-metastatic breast cancer cells. For instance, TGFβ-mediated activation of the AKT signaling was shown to be crucial for breast cancer cell proliferation and cancer stem cell properties selectively in the microenvironment of bone metastases [66]. More precisely, osteoblast-derived TGFβ1 stimulates, among others, the AKT/NF_K_B axis and, therefore, enhances the transmembrane adhesion receptor integrin β1 and β3-mediated migration of breast cancer cells [67]. AKT activation, due to receptor tyrosine kinase transactivation, was shown to inhibit the FAS-associated factor 1 (FAF1)-mediated destabilization of the TGFβ receptor 2 (TβRII) in breast cancer cells. The accumulation of TβRII in these cells results in an increased response to TGFβ and, thus, in a higher bone metastasis [68]. Additionally, TGFβ in conditioned medium from breast cancer cells was shown to inhibit the differentiation and mechanosensing properties of bone cells via the PI3K/AKT axis [69]. Interestingly, Hiraga et al. reported that bone-derived IGF-1 promotes bone metastasis and survival as well as proliferation of bone-metastatic breast cancer cells via an insulin-like growth factor 1 receptor (IGF-1R)/AKT/NF_K_B pathway independently of other bone-derived growth factors, such as TGFβ, fibroblast growth factor (FGF), or platelet-derived growth factor (PDGF) [70]. Consequently, inhibition of IGF-1R leads to a reduced bone metastasis of breast cancer cells due to inhibition of the AKT signaling [71]. Additionally, the IGF-1R inhibitor PQIP diminishes the breast cancer cell-induced osteolysis in a bone metastasis mouse model by inhibiting the IGF-1/IGF-1R/AKT axis-dependent osteoclast formation [72]. The IGF-1Rβ/AKT axis was shown to get activated in bone-seeking breast cancer cells after the knockdown of the transcription factor of osteogenic differentiation, namely runt-related transcription factor 2 (RUNX2). This increase in AKT activity is accompanied by an impaired early metastatic dissemination to the bone but induces the outgrowth of bone metastases in later stages [73]. In contrast, Cohen-Solal et al. suggested a positive feedback loop between RUNX2 and AKT in breast cancer cells. In this model, RUNX2 gets activated by AKT, and RUNX2, in turn, activates AKT in part via mTORC2 [74]. Further bone-derived soluble factors cooperate with tumor-derived laminin 511, an adhesive and pro-migratory substrate for tumor cells, to facilitate migration and invasion of bone-metastatic breast cancer cells via an upregulation of the AKT signaling and MMP9. Consistently, pretreatment of bone-metastatic breast cancer cells with an AKT1/2-inhibitor leads to a diminished bone metastasis [75]. Knockdown of the c-Jun N-terminal kinase 2 (JNK2) leads to a diminished breast cancer metastasis to lung and bone as well as to a reduced pro-osteolytic RANKL expression by the breast cancer cells. Mechanistically, it was shown that knockdown of JNK2 as a common downstream effector of receptor tyrosine kinases attenuates phosphorylation of AKT after stimulation of the cells with hepatocyte growth factor (HGF), insulin, or heregulin [76].

Bone morphogenetic proteins (BMPs) play an important role in the bone microenvironment and physiologically regulate the formation of bone [77]. Ren et al. revealed that BMP9 suppresses the PI3K/AKT signaling-dependent proliferation, migration, and metastasis of HER2-positive breast cancer cells [78]. BMP9 further inhibits the secretion of RANKL from bone marrow-derived mesenchymal stem cells co-cultured with breast cancer cells. The decrease in secreted RANKL levels results in decreased pAKT levels in the co-cultured breast cancer cells and, consequently, in decreased breast cancer cell migration in vitro and bone resorption in vivo [79]. In contrast, BMP2 is known to promote bone metastasis by EMT in breast cancer. Mechanistically, Huang et al. observed a BMP2-mediated phosphorylation and degradation of the retinoblastoma-associated protein (Rb) via the AKT pathway. Downregulation of the tumor suppressor Rb and, in turn, upregulation of the cell adhesion protein CD44 promotes EMT in breast cancer cells [80]. 

Besides the important role of AKT in bone-metastatic breast cancer cells themselves, several studies investigated the role of AKT in the crosstalk between breast cancer cells and cells of the bone microenvironment. Sophocleous et al. found that conditioned medium from breast cancer cells stimulates AKT phosphorylation in osteoclasts and, thus, promotes osteoclastogenesis. This effect could be further promoted by the serum calcium-regulating parathyroid hormone (PTH) or by an agonist of the cannabinoid type 2 receptor (CB2) [81]. Additionally, bone colonization of breast cancer cells upregulates the expression of the interferon-γ-inducible chemokine C-X-C motif chemokine 10 (CXCL10) in macrophages. After binding to its receptor C-X-C chemokine receptor type 3 (CXCR3), CXCL10 activates the AKT-mediated RANKL expression in osteoblast and, thus, facilitates RANKL-mediated osteoclast formation and osteolysis [82]. When breast cancer cells overexpress regucalcin, a calcium-dependent suppressor of intracellular signaling, they exhibit a reduced AKT signaling and suppressed osteoclastogenesis when co-cultured with bone marrow cells [83]. Mitochondrial fission in tumor cells is known to reduce the efficiency of oxidative phosphorylation and suppress tumor migration. Breast cancer cells with predominant mitochondrial fission display a reduced AKT activation accompanied by a reduction in bone metastasis and cancer-induced osteolysis [84]. Breast cancer cells expressing the chondroitin sulfate proteoglycan member versican G3-domain exhibit enhanced migration and invasion to primary bone stromal cells or osteoblasts in part via the epidermal growth factor receptor (EGFR)/AKT axis. Additionally, breast cancer-derived versican G3-domain suppresses the survival and differentiation of osteoblasts, suggesting a further contribution to osteolytic breast cancer bone metastasis [85]. Normanno et al. observed that inhibition of EGFR-mediated AKT activation in bone marrow stromal cells reduces the expression of osteoclastogenesis-inducing RANKL in these cells [86]. The pro-inflammatory Oncostatin M, which was shown to be produced by breast cancer cells, facilitates the pro-tumoral M2 polarization of macrophages via a mTORC2/AKT signaling axis. The M2 polarization of macrophages was shown to promote breast cancer metastasis to lung, liver, and bone in this study [87]. 

The efficacy of inhibitors in the treatment of breast cancer bone metastasis was studied in a plethora of studies, and it was shown that some of the inhibitors mediate their effect via the inhibition of AKT. Besides an inhibitory effect on proliferation, migration, and invasion of breast cancer cells, the PI3K/mTOR-inhibitor PKI-402 was also shown to inhibit RANKL-mediated osteoclastogenesis and, thus, tumor-induced osteolysis through suppression of the PI3K/AKT/mTOR pathway [88]. Additionally, Wang et al. observed a decreased AKT activation in breast cancer cells as well as osteoclasts after treatment with Raddeanin A, the bioactive constituent of anemone raddeana regel. This results in a reduction of RANKL-mediated osteoclast formation, tumor-induced osteolysis, tumor growth, and invasion [89]. The artemisia herb derivate Dihydroartemisinin inhibits osteoclast differentiation as well as proliferation, migration, and invasion of breast cancer cells in vitro through suppression of AKT phosphorylation in osteoclasts and breast cancer cells. Consequently, Dihydroartemisinin reduces breast cancer bone metastasis and breast cancer-mediated osteolysis in vivo [90]. Asperolide A, a marine-derived agent, attenuates breast cancer-induced osteolysis through the inhibition of osteoclast differentiation and bone resorption by blocking the PI3K/AKT/mTOR/c-FOS/NFATc1 axis. NFATc1 is known to directly regulate osteoclast-specific genes [91]. The natural coumarin Wedelolactone is another substance that inhibits breast cancer-induced osteoclastogenesis via a suppression of the AKT signaling in osteoclasts [92]. The inhibition of hyaluronan synthesis by 4-methylumbelliferone is due to a suppressed phosphorylation of AKT, and treatment of breast cancer cells with 4-methylumbelliferone leads to smaller osteolytic lesions in a mouse model [93]. The bisphosphonate zoledronic acid inhibits the secretion of the chemotactic cytokine RANTES and the pro-inflammatory cytokine interleukin-6 (IL-6) from bone-marrow-derived mesenchymal stem cells accompanied by a diminished AKT activation. RANTES and IL-6 usually promote breast cancer progression in bone and tumor-induced osteoclast formation via activation of the AKT signaling [94,95]. Furthermore, zoledronic acid suppresses breast cancer growth through inhibition of the AKT/forkhead box protein O3a (FOXO3a)-mediated expression of the extracellular matrix-associated protein CCN family member 1 (CCN1). Hence, pAKT levels are decreased after treatment with zoledronic acid [96]. It was further shown that bisphosphonates inhibit the vascular endothelial growth factor (VEGF)-mediated angiogenesis of breast cancer cells via suppression of the IGF-1/AKT/HIF-1α/VEGF axis [97]. Hu et al. observed that the combination of an IGF-1R inhibitor with zoledronic acid synergistically inhibits bone metastasis [71]. Zoledronic acid further regulates several microRNAs (miR), for example, a downregulation of miR-21, which are involved in the regulation of the PI3K/AKT signaling pathway [98,99]. These findings highlight the role of AKT in the treatment of breast cancer bone metastasis.

## 3. Prostate Cancer

Bone metastases frequently occur in patients with prostate cancer and mainly present as osteoblastic lesions but also possess osteolytic elements due to osteoclast stimulation. Hence, osteoblast- as well as osteoclast-stimulating factors play a crucial role in prostate cancer bone metastasis. Once bone metastases emerge, the survival rate of prostate cancer patients decreases dramatically, and complications, such as bone pain or pathological fractures, emerge [2,14,15,17].

The AKT signaling was found to be higher in bone-metastatic prostate cancer cells compared to non-bone-metastatic cells. An elevated AKT activation was also observed in probes of human bone metastases of prostate cancer in comparison to the corresponding primary tumor [100]. In an experimental setting, the bone marrow-derived extracellular matrix was shown to activate, among other kinases, the AKT kinase, resulting in an enhanced survival of prostate cancer cells [101]. In a subcutaneous mouse model, AKT was further suggested to drive EMT of prostate cancer cells and, thus, the dissemination to the bone marrow in the initiation stage [102]. Additionally, Mimeault et al. revealed that bone metastases from prostate cancer patients displayed a positive staining for pAKT serine 473 in 80% of the cases, which was higher than in the primary prostate cancer samples with 66% [103]. More precisely, osteolytic lesions of human prostate cancer show enriched pAKT levels in the tumor area and associated macrophages, whereas osteoblastic lesions were shown to possess higher phospho-signal transducer and activator of transcription 3 (pSTAT3) levels [104]. This suggests that a differential regulation of AKT and STAT signaling predicts either an osteolytic or an osteoblastic phenotype of bone metastases in prostate cancer.

**Figure 2:**
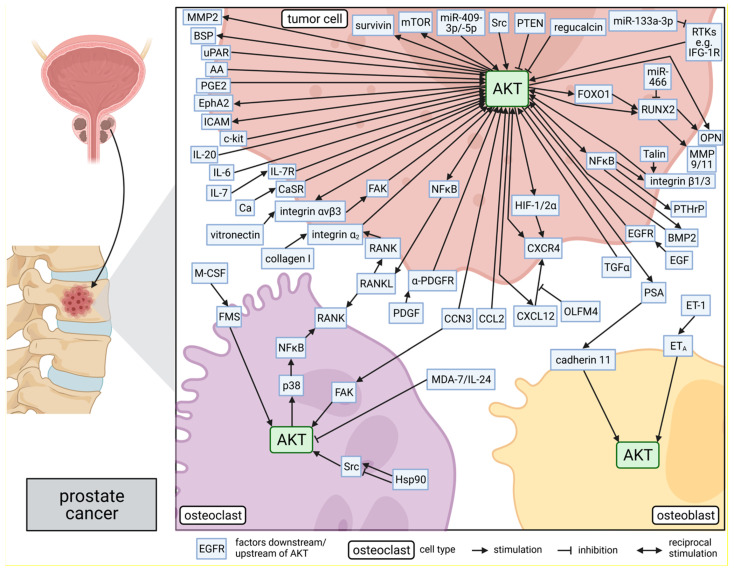
Role of AKT in molecular mechanisms of prostate cancer bone metastasis: The side panel on the left side shows schematically the process of prostate cancer metastasis to the bone. AKT is involved in several bone metastasis-promoting signaling pathways within prostate tumor cells, osteoclasts, and osteoblasts, as shown in the main part of the figure on the right. The position of the proteins indicates their subcellular location in the corresponding cells: intracellular, within the membrane, or secreted. Lines connecting the proteins represent either a promoting or suppressing interaction. This figure was created with BioRender.com.

Molecular mechanisms mediating the AKT-dependent bone-metastatic properties of prostate cancer cells were studied by several laboratories and are discussed in the following, as well as are presented in Figure 2.

As stated in the preceding section, the CXCL12/CXCR4 axis plays a crucial role in bone metastasis and the interaction between tumor cells and bone cells [60]. Bone marrow mesenchymal stem cell-secreted CXCL12 induces migration of prostate cancer cells via activation of AKT [105]. Overexpression of AKT1 in prostate cancer cells was shown to upregulate the CXCR4 expression and, therefore, promotes intraosseous tumor growth as well as the formation of metastases with an osteolytic phenotype. Consistently, loss of PTEN in prostate cancer cells enhances the AKT-mediated expression of CXCL12 and CXCR4 [106]. Furthermore, Chinni et al. revealed a CXCL12/CXCR4/AKT1/MMP9 axis in prostate cancer cells, and the resulting CXCL12-mediated AKT1 phosphorylation causes an increased migration as well as invasion [107]. Prostate cancer cells expressing the secreted glycoprotein Olfactomedin 4 (OLFM4) show a reduced bone metastasis due to an inhibited CXCL12-mediated AKT phosphorylation [108]. Hypoxia and the hypoxia-induced expression of the transcription factors HIF-1α or HIF-2α promote bone metastasis of prostate cancer, for example, via upregulation of CXCR4. The expression and stabilization of HIF-α proteins were shown to be dependent on AKT signaling. Consistently, stimulation of prostate cancer stem cells with CXCL12 was shown to induce AKT-mediated bone metastasis [62].

C-C motif chemokine 2 (CCL2) is another cytokine, which plays a crucial role in the bone microenvironment and is produced by bone cells as well as prostate cancer cells. CCL2 was shown to promote migration and invasion of prostate cancer cells and to facilitate the formation of osteoclasts. These effects were suggested to be partly AKT-dependent because CCL2 stimulates proliferation and survivin-mediated survival via the AKT pathway [109]. Furthermore, Graham et al. revealed that the PI3K/AKT signaling in prostate cancer cells is involved in the NF_K_B-mediated expression and secretion of BMP2, which stimulates the formation of bone metastases through the induction of osteoblast differentiation [110]. Osteoblast-secreted BMP2, in turn, was found to activate the AKT/NF_K_B axis in prostate cancer cells, leading to enhanced migration of the cells via an activation of the transmembrane adhesion receptors integrin β1 and β3 [111]. Bone metastases of prostate cancer in humans express higher levels of the extracellular matrix-associated protein CCN family member 3 (CCN3) compared to the primary tumor. Prostate cancer cells with a high CCN3 expression were shown to preferably metastasize to the bone [112]. Consistently, a knockdown of CCN3 was found to decrease bone metastasis of prostate cancer cells since CCN3 upregulates the expression of the pro-migratory intercellular adhesion molecule 1 (ICAM-1) and promotes migration via AKT, NF_K_B, and other pathways [113]. Cooper et al. reported that expression of the integrin αvβ3 in prostate cancer is associated with bone metastasis since the transmembrane adhesion receptor integrin αvβ3 mediates migration and adhesion to the bone matrix. Activation of integrin αvβ3 by binding, for example, to vitronectin, a glycoprotein of the bone, results in a focal adhesion kinase (FAK)-mediated AKT activation as a possible mediator for enhanced migration and bone metastasis [114]. Additionally, Pola et al. showed that integrin αvβ3 is able to activate the AKT/mTOR axis independently of FAK activation to facilitate protein synthesis under hypoxic conditions, thus promoting the invasion of prostate cancer cells [115]. Consistently, inhibition of AKT diminishes the expression of bone sialoprotein (BSP), OPN, pro-invasive matrix metalloproteinase-2 (MMP2), and αvβ3-integrin in prostate cancer cells, which are all proteins associated with the occurrence of bone metastases [116].

In osteoblasts, the transcription factor RUNX2 is physiologically responsible for the expression of genes, which are associated with osteoblastic differentiation [18]. Prostate cancer patients with lymph node and bone metastases display a low PTEN expression and, therefore, a high AKT activation, which is correlated with the overexpression of RUNX2 in the tumor cells. RUNX2, in turn, plays an important role in prostate cancer cells, and its expression was shown to be regulated via a PTEN/AKT/forkhead box protein O1 (FOXO1) axis [117]. Furthermore, Cohen-Solal et al. reviewed a reciprocal regulation of RUNX2 and AKT signaling in prostate cancer. More precisely, AKT promotes the RUNX2 expression and, therefore, the expression of RUNX2-dependent genes, such as the pro-invasive and pro-angiogenic MMP9, whereas RUNX2, in turn, facilitates AKT phosphorylation [74]. Furthermore, the pro-inflammatory arachidonic acid-derived prostaglandin E2 (PGE2) was shown to enhance the expression of the bone metastasis-promoting proteins MMP2, MMP9, RANKL, and RUNX2 in prostate cancer cells, partly via the PI3K/AKT pathway [118]. Downregulation of the microRNA miR-466 also contributes to bone metastasis of prostate cancer. MiR-466 targets RUNX2 and, therefore, leads to a suppression of RUNX2-related gene expression, such as AKT, OPN, matrix metalloproteinase-11 (MMP11) in prostate cancer cells [119]. Upregulation of another microRNA, miR-409-3p/-5p, was found in bone metastatic prostate cancer cells and is correlated with AKT activation. Consequently, inhibition of miR-409-3p/-5p results in a decreased bone metastasis [120].

Tawadros et al. observed that arachidonic acid (AA) stimulates the AKT-mediated ligand-independent activation of ephrin type-A receptor 2 (EphA2), a pro-invasive tyrosine kinase receptor. Therefore, AA-induced AKT activation increases invasion of prostate cancer cells through bone marrow endothelium [121]. Lower expression of the intracellular signaling suppressor regucalcin was found in metastatic tissue of prostate cancer patients compared to the primary tumor. Experimental overexpression of regucalcin in prostate cancer cells suppresses inter alia PI3K and AKT levels as well as diminishes osteoblast-mediated mineralization and osteoclastogenesis when co-cultured with pre-osteoblastic or -osteoclastic cell lines [122]. Ablation of the integrin-adaptor protein Talin-1 leads to a reduced activation of integrin β1 and, therefore, to a lower colonization of prostate cancer to the bone in vivo, which is accompanied by a decreased pAKT level [123]. The receptor tyrosine kinase c-kit is also involved in migration, invasion, and intraosseous tumor growth of prostate cancer cells and mediates its effect in a PI3K/AKT-dependent manner [124].

Dolloff et al. revealed that human bone marrow activates the PI3K/AKT signaling via a PDGF-mediated platelet-derived growth factor receptor α (α-PDGFR) transactivation selectively in bone-metastatic prostate cancer cells but not in non-bone-metastatic cells. Thus, the bone-metastatic potential of prostate cancer cell lines correlates with the expression of the receptor tyrosine kinase α-PDGFR and, thereby, with a stronger AKT activation [125,126]. An overexpression of RANKL in prostate cancer cells growing in a 3D culture leads to enhanced binding to collagen I through an increased expression of α2 integrin and subsequent activation of the FAK/AKT axis. As collagen I is the most abundant ECM protein in the bone, these findings suggest a facilitated adhesion to the bone matrix [127]. Interestingly, transforming growth factor α (TGFα) is able to stimulate the RANKL expression selectively in late-stage prostate cancer cells and, thus, induces osteoclast differentiation. Mechanistically, TGFα, a member of the epidermal growth factor (EGF) family, was shown to bind to the EGFR leading to a significant AKT activation in the late-stage prostate cancer cells, whereas stimulation of early-stage prostate cancer cells with EGF diminishes AKT activation. These findings suggests a stage- and ligand-dependent role of the EGFR/AKT axis in prostate cancer and highlights a role of AKT in the RANKL expression [128]. The pro-metastatic growth factor interleukin-7 (IL-7) was shown to stimulate the AKT signaling, among other pathways, in prostate cancer cells. Additionally, the overexpression of the interleukin-7 receptor (IL-7R) in prostate cancer cells increases the bone metastasis in a mouse model [129]. Further, the pro-inflammatory cytokine IL-6 was shown to activate AKT signaling and to promote bone metastasis of prostate cancer cells [95]. Liao et al. revealed that elevated extracellular calcium, like it is present in the bone microenvironment, selectively induces proliferation and metastasis of bone-metastatic but not of non-metastatic prostate cancer cells. This effect is mediated by a calcium sensing receptor (CaSR)-mediated AKT activation in these cells [130]. The microRNA miR-133a-3p is downregulated in human bone metastases of prostate cancer. More particularly, miR-133a-3p suppresses the AKT signaling by targeting multiple receptor tyrosine kinases, such as EGFR and IGF-1R. Thus, a lower level of miR-133a-3p is associated with a shorter bone metastasis-free survival in prostate cancer patients [131]. 

In addition to the well-characterized role of AKT in prostate cancer cells themselves, several laboratories investigated the effect of AKT on the crosstalk between prostate cancer cells and bone cells. Although prostate cancer causes mainly osteoblastic lesions via activation of osteoblasts, stimulation of osteoclasts by prostate cancer-derived factors further promotes the progression of prostate cancer bone metastases. Therefore, the crosstalk of prostate cancer cells with osteoblasts as well as with osteoclasts is important for bone metastasis of prostate cancer cells [132]. Zhu et al. reported that the expression of a constitutive phosphorylated AKT in prostate cancer cells leads to enhanced NF_K_B-mediated upregulation of RANKL, PTHrP, and BMP2. These proteins are known to be crucial for the crosstalk between tumor cells and osteoblasts as well as osteoclasts. Furthermore, coculturing prostate cancer cells and osteoclasts results in an enhanced activation of AKT and an increased proliferation of both cell lines, suggesting a crucial role of AKT for the crosstalk between these both cells [133]. AKT, in turn, was shown to promote the differentiation of osteoclasts [42]. Pradhan et al. revealed a dual role of AKT in bone metastasis of prostate cancer. On the one hand, inhibition of AKT with the therapeutic anticancer cytokine MDA-7/IL-24 in osteoclast precursor cells inhibits osteoclast differentiation. On the other hand, overexpression of a constitutively active AKT in prostate cancer cells results in enhanced bone metastasis in a mouse model accompanied by a higher osteoclastic activity. These effects could be abrogated by treating the mouse with the AKT-inhibitory MDA-7/IL-24 [134]. Additionally, prostate cancer-secreted CCN3 was shown to mediate RANK upregulation in osteoclast precursors via the FAK/AKT/mitogen-activated protein kinase 11 (p38)/NF-κB axis and, thus, promotes the RANKL-dependent osteoclastogenesis and tumor-induced osteolysis [112]. On the other hand, the prostate cancer-secreted serine protease prostate-specific antigen (PSA) induces osteogenic differentiation of mesenchymal stem cells via a cadherin 11/AKT axis and, thus, contributes to the formation of osteoblastic lesions [135]. In summary, much data exists on the role of AKT in the interaction between prostate tumor cells and osteoclasts. Although prostate cancer preferably causes osteoblastic lesions, the impact of AKT in the interaction between prostate cancer cells and osteoblasts remains mainly unidentified.

In the following, we discussed studies that linked the inhibitory effect of several inhibitors on bone metastasis of prostate cancer cells to an inhibition of the AKT signaling. For instance, blocking the PI3K/AKT axis in prostate cancer cells by the administration of the PI3K inhibitor ZSTK474 prevents bone metastasis in vivo via downregulation of the pro-invasive MMP9 [136]. The dual PI3K/mTOR inhibitor X480 was shown to inhibit bone metastasis and tumor-induced osteolysis in vivo as well as inhibits prostate cancer-induced osteoclastogenesis and stimulates osteoblast activity in vitro [100]. Watanabe et al. showed that the inhibition of the vascular endothelial growth factor receptor (VEGFR) and the hepatocyte growth factor receptor MET with the tyrosine kinase inhibitor TAS-115 diminishes the macrophage colony-stimulating factor (M-CSF)/FMS-dependent AKT activation in osteoclasts. Since M-CSF mediates osteoclast differentiation via its receptor FMS and subsequent AKT activation, TAS-115 leads to a reduced NF_K_B-mediated osteoclast formation and, thus, prostate cancer-induced bone resorption in a xenograft mouse model [137]. In addition, the quinonemethide triterpenoid compound Pristimerin was shown to suppress VEGF-induced vasculogenesis in a prostate cancer bone metastasis model by blocking the AKT signaling in bone marrow-derived endothelial progenitor cells [138]. Inhibition of the pro-inflammatory interleukin-20 (IL-20) suppresses prostate cancer-induced osteolysis in a xenograft mouse model, at least in part, via the AKT pathway [139]. The natural agents isoflavone and 3,3′-Diindolylmethane inhibit the differentiation of osteoblasts and osteoclasts in a prostate cancer co-culture system, in part, via a downregulated AKT in the context of the homeobox protein Nkx-3.1 (NKX3-1)/AKT/cyclin-dependent kinase inhibitor 1B (p27) axis. This results in an upregulation of the tumor suppressor p27, an inhibitor of the cell cycle progression [140]. Furthermore, inhibition of the chaperon protein heat shock protein 90 (Hsp90) with 17-N-Allylamino-17-demethoxygeldanamycin (17AAG) was shown to activate AKT in an Src-dependent way in osteoclasts and, thus, promotes osteoclastogenesis as well as increases the intraosseous tumor growth of prostate cancer cells after intratibial inoculation [141]. In contrast, the Hsp90 inhibitor PF-04928473 inhibits osteoclastogenesis via inhibition of Src activation and suppresses prostate tumor growth partly by downregulating AKT [142]. The underlying mechanisms of these contradictory findings remain unclear. Combined inhibition of IGF-1R and the Src family non-receptor tyrosine kinases leads to an effective suppression of AKT1 and AKT2 activation and consequently to a decreased intratibial tumor growth of prostate cancer cells [143]. The Src/Abl kinase inhibitor bosutinib was shown to inhibit the phosphorylation of AKT among other kinases as well as the expression of pro-osteolytic proteins in prostate cancer cells, such as MMP9, the pro-metastatic glycoprotein urokinase plasminogen activator surface receptor (uPAR), or the chemotactic cytokine interleukin-8 (IL-8). Since these proteins play a pivotal role in the development of skeletal metastases in prostate cancer, bosutinib prevents the formation of skeletal lesions and osteolysis in a bone metastasis mouse model [144]. Rabbani et al. observed that the uPAR inhibitor ATN-658 blocks the formation of intraosseous metastases of prostate cancer cells partly via inhibition of AKT phosphorylation [145]. Similar to breast cancer, the hyaluronan synthesis inhibitor 4-methylumbelliferone inhibits the formation of bone metastases of prostate cancer partly by blocking the AKT pathway [146]. In addition to its inhibitory effect on osteoclastogenesis, the salicylanilide derivative LCC03 inhibits the intraosseous growth of prostate cancer cells by inducing autophagy via a reduction of the AKT signaling [147]. Prostate cancer expressing endothelin 1 (ET-1) was shown to preferentially metastasize to the bone because ET-1 binds to the endothelin receptor A (ET_A_) on bone marrow stromal cells and, therefore, causes proliferation and activation of bone-forming osteoblasts. As a consequence, a combinatorial treatment with the chemotherapeutic agent docetaxel and the ET_A_ inhibitor ABT-627 leads to a reduced prostate tumor growth within the bone marrow of mice via suppression of the AKT/NF_K_B axis [148]. These data suggest an important role of AKT inhibition in prostate cancer bone metastasis.

## 4. Lung Cancer

**Figure 3:**
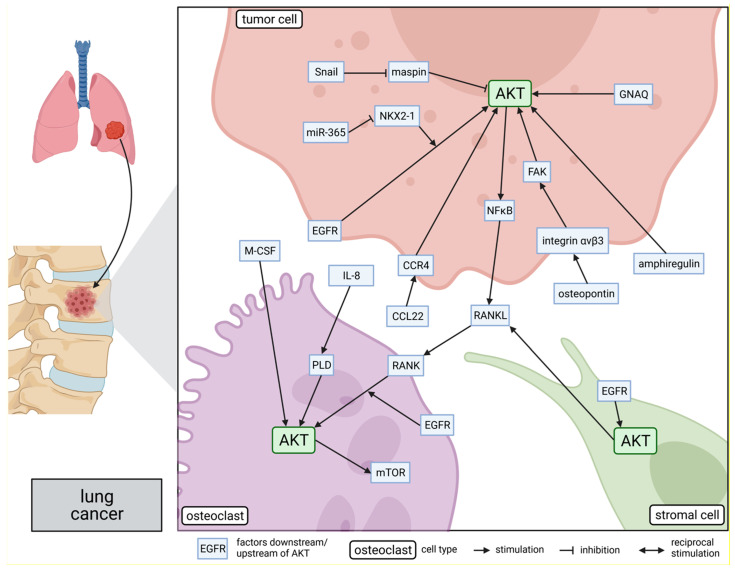
Role of AKT in molecular mechanisms of lung cancer bone metastasis: The side panel on the left side shows schematically the process of lung cancer metastasis to the bone. AKT is involved in several bone metastasis-promoting signaling pathways within lung tumor cells, osteoclasts, and stromal cells, as shown in the main part of the figure on the right. The position of the proteins indicates their subcellular location in the corresponding cells: intracellular, within the membrane, or secreted. Lines connecting the proteins represent either a promoting or suppressing interaction. This figure was created with BioRender.com.

Similar to breast and prostate cancer, bone is also a preferential distant site for lung cancer metastasis [3]. Metastases of lung cancer predominantly cause osteolytic lesions and lead to a higher morbidity and mortality [149,150]. A few studies investigated molecular mechanisms mediating an AKT-dependent progression of lung cancer bone metastases and an AKT-dependent interaction of lung cancer cells with bone cells within a vicious cycle of osteolytic bone metastasis. These studies are summarized in Figure 3 and are discussed in the following.

Migration of lung cancer cells was shown to be stimulated by osteopontin, which is a frequently expressed molecule in the ECM of bone. Mechanistically, osteopontin binds to the transmembrane adhesion receptor integrin αvβ3 and, thus, activates the pro-metastatic FAK/PI3K/AKT/NF_K_B axis in these cells [151]. Liu et al. observed that a low serum level of the microRNA miR-365 in patients with lung cancer is associated with the occurrence of bone metastases. Downregulating miR-365 in non-small-cell lung cancer cells was shown to upregulate the EGFR/PI3K/AKT axis through the pro-metastatic transcription factor homeobox protein Nkx-2.1 (NKX2-1) [152]. 

Nakamura et al. revealed that the osteoclast-derived chemotactic cytokine C-C motif chemokine 22 (CCL22) activates the AKT pathway in lung cancer cells via the C-C chemokine receptor type 4 (CCR4) and, thus, promotes bone metastasis. Consistently, CCL22 producing osteoclasts and CCR4 expressing lung cancer cells were shown to be colocalized in bone metastases [153]. The knockdown of the inhibitory guanine nucleotide-binding protein G(q) subunit alpha (GNAQ) in lung cancer cells promotes the RANKL expression and, therefore, osteoclastogenesis due to an enhanced AKT/NF_K_B signaling [154]. Moreover, conditioned medium of lung cancer cells, as well as treatment with M-CSF/RANKL, were shown to induce osteoclastogenesis through activation of the AKT/mTOR axis [155]. Furthermore, the lung cancer-derived pro-inflammatory chemokine IL-8 induces osteoclastogenesis via the phospholipase D (PLD)/AKT axis. Consistently, blocking of AKT was shown to suppress osteoclast differentiation [156]. The EGFR tyrosine kinase inhibitor afatinib is already used for the therapy of non-small cell lung cancer. It was further shown that afatinib suppresses osteoclast formation and, therefore, bone resorption by inhibiting the RANKL-induced AKT activation in pre-osteoclasts [157]. Additionally, blocking the EGFR/AKT axis using the EGFR-inhibitor gefitinib in bone marrow stromal cells suppresses the RANKL production of these cells. The reduced RANKL levels result in a further diminished formation of osteoclasts [86]. Tumor-associated dendritic cells-derived amphiregulin, a growth factor of the epidermal growth factor family, mediates an osteolytic bone metastases phenotype in lung cancer cells, accompanied by an activation of AKT and STAT3 [158]. 

The bioflavonoid compound quercetin was shown to suppress motility and bone metastasis of lung cancer cells in a mouse model through downregulation of the EMT-associated transcription factor Snail followed by a maspin-mediated inhibition of AKT activity [159].

## 5. Other Cancer Entities

In opposition to the extensive data on bone metastasis of breast, prostate, and lung cancer, only little is known about the role of AKT in bone metastasis of other cancer entities, such as malignant melanoma, renal cell carcinoma, bladder cancer, hepatocellular cancer, colorectal cancer, gastric cancer, oral squamous cell carcinoma, or thyroid cancer. The scarce data on these cancer entities are summarized below.

### 5.1. Malignant Melanoma

In malignant melanoma cells, AKT and extracellular signal-regulated kinase (ERK) signaling are involved in the bone metastasis-promoting effect of the master transcription factor of osteogenic differentiation RUNX2. Inhibition of AKT in this tumor entity reduces the levels of pro-metastatic PTHrP and Osteonectin as well as Osteocalcin, which are RUNX2-dependent proteins [160]. Additionally, RUNX2 was shown to upregulate the focal adhesion-associated protein kinase FAK, which, in turn, is able to activate AKT [74]. The growth of malignant melanoma cells in mouse bone marrow promotes the expression of the interferon-γ-inducible chemokine CXCL10 by the host bone marrow. CXCL10, in turn, upregulates the RANKL expression of osteoblasts via an AKT activation and, thereby, promotes osteoclast formation and tumor-induced osteolysis [82].

### 5.2. Renal Cell Carcinoma

Renal cell carcinoma cells from patients with bone metastases exhibit an enhanced migration and adhesion to collagen I and fibronectin, which are abundant in the bone matrix. The enhanced migration and adhesion in these cells are accompanied by an elevated AKT activation compared to non-metastatic cells [161]. In renal carcinoma cells, the calcium sensing receptor (CaSR) was shown to induce proliferation, migration, and adhesion towards endothelial cells, collagen I, and fibronectin in vitro as well as the formation of bone metastases in vivo. This is partly due to a CaSR-mediated activation of AKT as a response to the high calcium concentrations within the bone microenvironment [162,163].

### 5.3. Bladder Cancer

Wu et al. showed that bladder cancer cells downregulate AKT activity after they colonized the bone. The reduced PI3K/AKT signaling, in turn, leads to a disinhibition of the AKT substrate GSK3β and consequently to a reduced β-catenin-mediated expression of the pro-invasive and EMT-promoting zinc finger E-box-binding homeobox 1 (ZEB1) protein. As a result, downregulation of AKT in bladder cancer cells, which have already spread to the bone, promotes mesenchymal-epithelial transition (MET) and, thus, growth of epithelial-like cancer cells in the bone marrow [164].

### 5.4. Hepatocellular Carcinoma

Bone marrow stromal cell-derived C-C motif chemokine 5 (CCL5), also known as the chemotactic cytokine RANTES, was shown to induce migration and invasion of hepatocellular carcinoma cells via the PI3K/AKT pathway and, therefore, could promote bone metastasis [165]. Furthermore, the long non-coding RNA zinc finger E-box binding homeobox 1 antisense 1 (lncZEB1-AS1) is associated with the bone metastasis of human hepatocellular carcinoma. Mechanistically, lncZEB1-AS1 was found to target the microRNA miR302b and, thus, increases the PI3K/AKT signaling in hepatocellular carcinoma cells [166].

### 5.5. Colorectal Cancer

Bao et al. revealed that the osteoblast-secreted pro-metastatic protein periostin activates the AKT signaling in colon cancer cells by binding to integrin αvβ3, which is a transmembrane adhesion receptor associated with bone metastasis. The activation of AKT was further shown to enhance the survival of colon cancer cells, suggesting a promoting role in the outgrowth of bone metastases [167].

### 5.6. Gastric Cancer

High levels of the osteoinductive cytokine BMP2 in the serum of patients with gastric cancer were shown to be associated with bone metastasis. Mechanistical studies revealed that stimulation of gastric cancer cells with BMP2 leads to an activation of the AKT/NF_K_B/MMP9 axis and, thus, promotes EMT, motility, and invasion of these cells. Blocking the AKT signaling reduces the BMP2-mediated invasiveness of gastric cancer cells [168,169].

### 5.7. Oral Squamous Cell Carcinoma

The stimulation of oral squamous carcinoma cells with lysophosphatidic acid results in an upregulated AKT-dependent secretion of the pro-inflammatory cytokines IL-6 and IL-8. Both cytokines, in turn, promote osteoclastogenesis directly as well as indirectly through upregulation of osteoblast-secreted RANKL, suggesting an important role in bone metastasis of oral squamous cell carcinoma [170].

### 5.8. Thyroid Cancer

Younes et al. revealed that the dual tyrosine kinase inhibitor AEE788 inhibits the phosphorylation of EGFR and VEGFR as well as the activation of the AKT signaling downstream in follicular thyroid cancer cells. This results in an impaired intraosseous growth of the tumor cells in a mouse model [171].

## 6. Discussion

In this review, we pointed out the importance of AKT signaling in bone metastasis of various cancer entities and the corresponding postulated underlying molecular mechanisms. To sum up, AKT in tumor cells is associated with the formation of bone metastases in vitro, in vivo, and in human specimen and is stimulated by a series of bone-derived growth factors or cytokines via their corresponding receptors. The activated AKT kinase in the tumor cells mediates its bone metastasis-promoting effect through distinct signaling pathways, inter alia via upregulation of transcription factors and, thus, expression of bone cell-stimulating factors. Additionally, the stimulation of bone cells, such as osteoblasts or osteoclasts, by tumor cell-derived factors also activates the AKT pathway in the bone cells and, thus, promotes their differentiation and activity. Consistently, direct or indirect inhibition of AKT signaling was shown to suppress bone metastasis of various solid tumors. The here discussed data on mouse models and clinical studies evaluating the role of AKT in bone metastasis of breast, prostate, and lung cancer are summarized in Tables S1 and S2. Taken together, AKT plays a crucial role in several parts of the process of bone metastasis, including homing to the bone, survival within the bone microenvironment, and especially the crosstalk between tumor cells and bone cells.

In the following, the similarities and differences of the role of AKT in bone metastasis of breast, prostate, and lung cancer will be discussed. As stated above, several growth factors and cytokines, which are secreted from bone cells or are released during bone resorption, activates the AKT signaling in tumor cells and, thus, promotes the formation of bone metastases. The CXCL12/CXCR4 axis is one of the most important axes for the interaction between bone microenvironment and tumor cells and was shown to be important for AKT activation in breast as well as prostate cancer. In both cancer entities, AKT further promotes the expression of CXCR4 via stabilization of the transcription factor HIF-1α. However, HER2 was shown to promote the CXCL12/CXCR4/AKT axis only in breast cancer. Beyond that, the effect of some growth factors and cytokines on the AKT signaling is more important or was better studied in one of the three cancer entities. In breast cancer, the growth factors TGFβ and IGF play a particularly important role in the activation of AKT, whereas primarily EGFR was shown to promote AKT activation in bone metastases of lung cancer. In bone metastases of prostate cancer, by contrast, TGFα, PDGF, and interleukins were shown to play a predominant role in AKT activation. Moreover, BMP2 promotes bone metastasis of breast and prostate cancer via the AKT pathway, but BMP9 was shown to inhibit the AKT signaling in breast cancer and, thus, suppresses the formation of bone metastases. Integrins mediate the bone metastasis-promoting effect of bone ECM components primarily via a FAK-dependent AKT activation. Whereas integrin β1/3 is involved in AKT activation in breast and prostate cancer, the integrins αvβ3 and α2 only play an important role or were better studied in prostate cancer bone metastases.

The activated AKT kinase has an effect on several downstream effectors and signaling pathways, which facilitate bone metastasis of the tumor cells. For instance, AKT promotes the secretion of pro-invasive MMPs, especially MMP9, in breast as well as prostate cancer cells. The transcription factor RUNX2 is another effector, which was shown to be dependent on the AKT activation in both cancer entities. Interestingly, RUNX2 and AKT were shown to be reciprocally regulated in prostate cancer but not in breast cancer. Moreover, activation of RUNX2 by AKT has more impact and was better investigated in prostate cancer than in breast cancer since prostate cancer mainly causes osteoblastic lesions, and RUNX2 depicts a pro-osteogenic transcription factor. However, not all of the downstream effectors of AKT are involved in the bone metastasis of each of the three cancer entities. Secretion of PSA, which induces osteoblast activation, was shown to be an AKT-dependent effector exemplary in prostate cancer, whereas PTHrP was observed in breast as well as prostate cancer bone metastases.

AKT was also shown to be important in the activation and differentiation of osteoblasts and osteoclasts. The RANKL/RANK axis is regulated by AKT and/or activates the AKT signaling in osteoclasts after stimulation with RANKL, which is derived from breast, prostate, as well as lung cancer cells. Surprisingly, AKT activation is associated with osteolytic lesions in breast and lung cancer but also in prostate cancer, although prostate cancer cells predominantly cause osteoblastic lesions in patients. Furthermore, an osteoblastic phenotype of prostate cancer bone metastases is associated with an activation of the STAT3 signaling but not with AKT activation. Consistently, AKT was shown to be mainly involved in the secretion of osteolytic factors, such as PTHrP or MMP9, and in the RANKL-mediated activation of osteoclasts in prostate cancer.

The examination of inhibitors affecting the AKT signaling has a different focus in the publications depending on the investigated cancer entity. In breast cancer, the inhibitory effect of bisphosphonates on the AKT signaling was examined, whereas in lung cancer the influence of an EGFR inhibition on AKT was tested, and in prostate cancer the activation of AKT after inhibition of the pro-osteoblastic ET-1 receptor was the predominant subject of research papers. 

In summary, bone metastases of breast, prostate, and lung cancer share a few molecular mechanisms, which are responsible for the AKT activation in the tumor cells and the bone cells, as well as show a similar pattern of downstream effectors of an activated AKT in tumor and bone cells. Nevertheless, several mechanisms were only shown in one part of the cancer entities, suggesting also a tumor type-dependent function of the AKT signaling in bone metastasis.

Since bone metastases are often incurable and lacking a sufficient treatment, we propose on the basis of the data presented here to consider an AKT targeted therapy for bone metastasis of cancer. Plenty of AKT inhibitors already exist and were shown to have an anti-tumor effect in general in several tumor types, such as prostate or breast cancer [40,172,173,174,175,176]. Based on the studies discussed in this review, inhibition of AKT in bone metastases could suppress the stimulatory effect of bone-derived factors, such as TGFβ, EGF, and CXCL12, could prevent the secretion of bone cell-stimulating factors like RANKL, as well as could inhibit the activation of osteoclasts and osteoblasts. Thus, AKT inhibitors could impair the vicious cycle between tumor cells and bone cells and, as a consequence, could reduce the formation of bone metastases.

It can be further considered that treatment of bone metastases with an AKT inhibitor could be more effective in the initial stage of bone metastasis than in a later stage with already overt bone metastases. Hence, it is of particular interest to analyze the role of AKT in the initiation stage of bone metastasis of solid tumors as well as the contribution of AKT to the metastatic niche. However, only little is known about the explicit effect of AKT in the early phases of bone metastasis. As stated above, Shi et al. and Wang et al. reported a contribution of AKT in the initiation stage of breast and prostate cancer bone metastasis by facilitating the EMT in prostate cancer cells or mediating the stimulatory effect of E-N-heterotypic adherens junctions during the colonization of the osteogenic niche in breast cancer [54,102]. Furthermore, AKT could play a crucial role in the early phase of bone metastasis of breast and prostate cancer because the AKT signaling is strongly involved in different levels of the CXCL12/CXCR4 axis. The CXCL12/CXCR4 axis was shown to be important for the homing of breast and prostate cancer cells to the bone in the early phases of bone metastasis. CXCL12 serves as a chemoattractant for CTCs, which express its receptor CXCR4. Subsequently, the CXCL12/CXCR4 interaction drive the survival and adaptation to the requirements of the bone microenvironment after breast and prostate cancer colonized the bone marrow. Moreover, dormant disseminated tumor cells within the bone marrow are activated by growth factors, such as TGFβ, which are released during bone resorption within the premetastatic niche [7,177]. Since AKT was shown to be activated by TGFβ and to be involved in osteoclastogenesis, an important role of AKT in these processes of the initiation stage of bone metastasis could be presumed. In line with this hypothesis, Mimeault and Batra reported that AKT stabilizes HIF-1α and, thus, promotes HIF-1α-dependent CXCR4 expression in breast and prostate cancer cells. Consequently, the AKT/HIF-1α/CXCR4 axis could facilitate the colonization of the hypoxic endosteal niche during the initiation stage of bone metastasis [62]. Nevertheless, further research projects should clarify the role of AKT in the initiation stage of bone metastasis. These experiments should address the question, whether targeting AKT during the early phases of metastasis could prevent the formation of overt bone metastases.

Besides the direct and sole targeting of AKT in bone metastasis, a combination with an inhibitor of other molecular targets, which are involved in bone metastasis and are associated with the AKT signaling network, might be beneficial. For example, denosumab is a therapeutic anti-RANKL antibody, which is widely used in the therapy of osteolytic bone metastases as an alternative to bisphosphonates. Blocking the RANKL/RANK axis in osteoclasts suppresses the formation of tumor-induced osteolysis by preventing osteoclastogenesis and the resorptive activity of osteoclasts [178]. Since AKT was shown to mediate the effect of the RANKL/RANK axis in osteoclasts, a combination of an AKT inhibition with an anti-RANKL antibody might increase the efficacy of both drugs synergistically. The TGFβ-pathway is another possible target in the treatment of bone metastasis, and antibodies directed against TGFβ or against TGFβ receptors are subject of current studies [179]. Inhibition of the TGFβ signaling diminishes the stimulation of the tumor cells by bone matrix-derived TGFβ; thus, breaks the vicious cycle of bone metastasis and, as a consequence, reduces the formation of bone metastases [179,180]. The activation of the TGFβ receptor is also associated with an activation of the AKT signaling, and, thus, a combinatorial inhibition of AKT and TGFβ might be advantageous and could show synergistic effects. ET-1 antagonists and c-Src inhibitors depict further therapeutic targets in bone metastasis, which are associated with the AKT signaling and are currently under preclinical and clinical examination [181]. Whether a combination of an AKT inhibitor with an anti-TGFβ antibody, an anti-RANKL antibody, or an inhibitor of other molecular targets exerts additive or synergistic effects should be investigated in the future.

Several AKT inhibitors are already under examination in clinical trials for the general treatment of advanced cancer. For instance, the AKT inhibitor Ipatasertib was tested in the treatment of metastatic prostate cancer in combination with an anti-hormone therapy in a phase II study [174]. In other phase II clinical trials, the AKT inhibitors Ipatasertib and Capivasertib combined with paclitaxel show promising efficacy in metastatic breast cancer patients [175,176], and phase III studies analyzing these combinations in metastatic breast cancer are currently running [182,183]. Nevertheless, these studies analyze the inhibition of AKT in patients with metastatic cancer in general but do not distinguish between the efficacy of AKT inhibitors in patients with bone metastases compared to patients with non-bone metastases. The question of whether the efficacy of an AKT inhibition is different between patients with bone metastases and patients with non-bone metastases should be addressed in further clinical trials.

However, before the efficacy of AKT inhibitors in the treatment of bone metastases can be further analyzed in clinical trials, some remaining and striking questions need to be addressed in future experimental research projects. 

Firstly, the three AKT isoforms were shown to exert distinct and partly even opposing roles in tumorigenesis and cancer progression, as reviewed, for example, in breast cancer [184]. Furthermore, Li et al. observed an increased metastasis to the lung in mice inoculated with breast cancer cells after treatment with a panAKT inhibitor [185]. Our laboratory recently showed an increased bone metastasis without the promotion of osteolysis after generating a knockdown of AKT3 in bone-metastatic breast cancer cells [186]. Thus, further research has to clarify the role of the various AKT isoforms in bone metastasis of various cancer entities to assess the possibility of an isoform-specific AKT inhibition and to rule out possible adverse effects associated with panAKT inhibition. 

Secondly, it needs to be borne in mind that the inhibition of one player in a signaling network could lead to a counter-regulation by other players. One could mention here that the inhibition of mTOR with everolimus causes a hyperactivation of AKT in cancer cells, which could be overcome by a combination with a panAKT inhibitor [187,188]. Hence, potential counter-regulatory mechanisms, which could lead to an escape of bone-metastatic cancer cells from AKT inhibition, have to be identified or ruled out. 

Thirdly, the influence of an AKT inhibition on the physiological bone metabolism and homeostasis has to be examined. Because of the contribution of AKT in bone cells, especially in the RANKL-mediated osteoclastogenesis, an alteration of the physiological processes of bone remodeling after AKT inhibition seems possible and could cause severe adverse effects. 

If these and further emerging questions would be examined, the inhibition of AKT as a central player in bone metastasis could be further investigated in clinical trials and might overcome the lack of treatment options for patients with bone metastases in the future.

## 7. Conclusions

This review deals with the role of AKT in bone metastasis of various solid tumors. We pointed out that AKT is a central player in the vicious cycle of bone metastasis. More specifically, AKT is activated in the tumor cells by bone-derived factors, and, in turn, activates several signaling pathways as well as the secretion of bone cell-stimulating factors. These factors stimulate the AKT signaling in bone cells, such as osteoblast and osteoclast, promoting their activity and, thus, the formation of osteoblastic or osteolytic lesions. As a consequence, activation of AKT is associated with the formation of bone metastases in mouse experiments and in human specimen, and direct or indirect inhibition of AKT reduces the bone-metastatic capacity of several cancer entities. On that basis, AKT inhibitors depict a promising therapeutic target for the treatment of bone metastases and should be further investigated in experimental and clinical studies.

## Supplementary Materials

The following are available online at https://www.mdpi.com/article/10.3390/cancers13102287/s1, Table S1: Publications with mouse experiments evaluating the role of AKT in bone metastasis of breast, prostate, and lung cancer, Table S2: Clinical experiments evaluating the role of AKT in bone metastasis of breast, prostate, and lung cancer.
