# Supplementary material for: AKT in Bone Metastasis of Solid Tumors: A Comprehensive Review
Source: Cancers (Basel). 2021 May 11;13(10):2287. doi: 10.3390/cancers13102287 (PMC8151478; doi:10.3390/cancers13102287)
Supplement: Supplementary file 1 [file cancers-13-02287-s001.zip › cancers-1206298-supplementary.pdf]

cancers

MDPI

Supplementary Materials: AKT in Bone Metastasis of Solid Tumors: A Comprehensive Review

Nico Hinz and Manfred Jücker

Table S1. Publications with mouse experiments evaluating the role of AKT in bone metastasis of breast, prostate and lung cancer.

Type of solid tumor	Publication	Mouse model	Main findings
breast cancer	Buschhaus et al. 2020 [48]	NSG mice; MCF7 cells; femoral artery injection; bioluminescence imaging (BLI), FLIM or pHAEP microscopy	Combination of the AKT inhibitor MK2206 and the thioredoxin reductase inhibitor D9 reduces intraosseous tumor growth
	Liu et al. 2018 [49]	BALB/c mice; 4T1.2 cells; iliac artery injection; micro-CT analysis, mechanical testing and histological analysis	Fluphenazine and trifluoperazine inhibits intraosseous tumor growth via reduced pAKT levels
	Khotskaya et al. 2014 [50]	Athymic nude mice; MDA-MB-231 cells; tail vein injection; BLI imaging and X-ray analysis	The AKT/mTOR/S6K1 axis is hyperactivated in breast cancer cells harvested from bone metastases
	Wang et al. 2015 [54]	BALB/c mice; MCF7, MDA-MB-361, 4TO7 cells; Iliac artery injection; BLI and immunofluorescence analysis	E-N-heterotypic adherens junctions promote bone colonization via an activation of the AKT/mTOR pathway
	Sun et al. 2019 [56]	BALB/C mice; 4T1 cells; intratibial injection and mammary fat pad injection; IHC staining	CENPF and PTHrP expression are higher in bone metastases compared to mammary fat pad tumors (CENPF promotes PTHrP expression via PI3K/AKT/mTORC1 activation)
	Zeng et al. 2014 [59]	C57BL/6 nude mice; MDA-MB-231BA-rfp cells; tail vein injection; micro-PET analysis	Knockdown of CXCR4 inhibits bone metastasis via a suppression of the PI3K/AKT/MMP9 axis
	Zhang et al. 2009 [63]	Athymic or Bg-Xu mice; MDA-MB-231, BoM-1833, CN34 BoM2 cells; intracardiac, intratibial or mammary fat pad injection; BLI, X-ray and histological analysis Microarray gene expression of human breast cancer metastasis	Knockdown of c-Src reduces bone metastasis partly via a diminished CXCL12-dependent AKT activation
	Zhang et al. 2013 [64]	Bg-nu mice; various MDA-MB-231 and CN34 sublines; intracardiac injection or mammary fat pad injection; BLI; X-ray and histological analysis	Bone-metastatic cells show a high Src-dependent sensitivity for CXCL12- and IGF-1-mediated AKT activation and are selected by cancer-associated fibroblasts
	Fritsche et al. 2015 [65]	SCID mice; MDA-MB-231-BO cells; intracardiac injection; BLI, MRI, micro-Ct and histological analysis	Knockdown of TRAIL-R2 reduces bone metastasis via decreased levels of pAKT and CXCR4
	Futakuchi et al. 2019 [66]	BALB/c mice; Cl66M2 cells; subcutaneous injection and injection over the calvaria; IHC, tumor volume and histological analysis	TGFβ promotes the growth of bone metastases and the induction of cancer stem cells in the bone microenvironment partly via an AKT activation
	Xie et al. 2017 [68]	Nude or C57BL/6J mice; MDA-MB-231 cells; intracardiac injection; BLI	Tyrosine receptor kinase-mediated AKT activation reduces the FAF1-mediated destabilization of TβRII and therefore increases bone metastasis through an accumulation of TβRII
	Hiraga et al. 2012 [70]	Athymic nude and SCID mice; MDA-MB-231 cells; intracardiac injection; X-ray and histological analysis	IGF-1 promotes bone metastasis via an IGF-1R/AKT/NFκB axis independently of other bone-derived growth factors

	Hu et al. 2020 [71]	Nude mice; intracardiac inoculation; MDA-MB-231BO cells; BLI and histological analysis	Inhibition of IGF-1R reduces bone metastasis via diminished pAKT levels
	Logan et al. 2013 [72]	BALB/c mice; intratibial injection; 4T1 cells; micro-CT and histological analysis	The IGF-1R inhibitor reduces tumor-induced osteolysis via inhibition of the IGF-1/IGF-1R/AKT axis-dependent osteoclast formation
	Tandon et al. 2016 [73]	NSG mice; MDA-MB-231 cells; intracardial inoculation; BLI, micro-CT and X-ray analysis, harvesting of bone-seeking subpopulation	Knockdown of RUNX2 inhibits the early phase of bone metastasis but promotes the late outgrowth of bone metastases via an activation if the IGF-1Rβ/AKT axis
	Denoyer et al. 2014 [75]	BALB/c mice; 4T1BM2 cells; intracardiac injection; histological analysis and qPCR	Inhibition of AKT1 and AKT2 reduces bone metastasis
	Nasrazadani and Van Den Berg 2011 [76]	BALB/c mice; 4T1.2 cells; intracardiac injection; IHC	Knockdown of JNK2 diminished bone metastasis via a reduced AKT phosphorylation after stimulation with HGF, insulin or heregulin
	Ren et al. 2014 [78]	Nude mice; SK-BR-3 cells; subcutaneous injection; IHC and histological analysis	BMP9 suppresses tumor growth via an inhibited PI3K/AKT signaling
	Wan et al. 2014 [79]	Injection into human fetal femur fragments implanted into nude mice; MDA-MB-231 cells; IHC	BMP9 reduces the formation of osteolytic lesions via a decreased RANKL-mediated AKT phosphorylation
	Lee et al. 2012 [82]	Nude mice; MDA-MB-231 cells; intracardiac injection; X-ray and histological analysis	Knockdown of CXCR3 reduces bone metastasis via a lack of CXCL10-mediated AKT activation
	Humphries et al. 2020 [84]	NSG mice; MDA-MB-231 and SUM159 cells; intracardiac injection or femoral artery injection; BLI and micro-CT analysis	Mitochondrial fission decreases the formation of osteolytic bone metastases via a reduced AKT activation
	Shrivastava et al. 2019 [87]	BALB/c mice; 4T1 cells; mammary fat pad injection; X-ray, micro-CT and histological analysis	Oncostatin M promotes metastasis to bone, lung and liver via an mTORC2/AKT-mediated M2 polarization of macrophages
	Yuan et al. 2019 [88]	BALB/c mice; MDA-MB-231 cells; intratibial injection; micro-CT, histological, IHC and PCR analysis	The PI3K/mTOR-inhibitor PKI-402 inhibits the formation of osteolytic metastases via a suppression of the PI3K/AKT/mTOR axis
	Wang et al. 2018 [89]	BALB/c mice; MDA-MB-231 cells; intratibial injection; micro-CT and histological analysis	Raddeanin A inhibits the formation of osteolytic lesions via a reduction of pAKT levels
	Feng et al. 2016 [90]	BALB/c mice; MDA-MB-231 cells; intratibial injection; micro-CT, histological and IHC analysis	Dihydroartemisinin reduces the formation of osteolytic metastases via an inhibition of AKT
	Jiang et al. 2020 [91]	BALB/c mice; MDA-MB-231 cells; intratibial injection; micro-CT and histological analysis	Asperolide A attenuates breast cancer-induced osteolysis via an inhibition of the PI3K/AKT/mTOR/c-FOS/NFATc1 axis in osteoclasts
	Urakawa et al. 2012 [93]	BALB/c mice; MDA-MB-231 cells; intratibial injection; X-ray and histological analysis	4-methylumbelliferone attenuates the formation of osteolytic lesions via a suppressed AKT phosphorylation
	Tang et al. 2010 [97]	Nude mice; MCF-7 cells; subcutaneous injection; hemoglobin amount analysis	Clodornate inhibits angiogenesis via a suppression of the IGF-1/AKT/HIF-1α/VEGF axis
prostate cancer	Shi et al. 2018 [102]	BALB/c, SCID or C57BL/6J mice; PC-3, C4-2B or RM-1 cells; subcutaneous injection; BLI and subsequent array analysis	AKT promotes EMT and therefore colonization of prostate cancer cells to the bone marrow in the initiation stage
	Conley-LaComb et al. 2013 [106]	SCID mice; DU145 cells; intratibial injection; histological analysis	Overexpression of AKT1 leads to higher intraosseous tumor growth via an upregulation of CXCR4

Chinni et al. 2006 [107]	Injection into human fetal femur fragments implanted into SCID mice; PC-3 cells; IHC analysis	CXCL12 causes a phosphorylation of AKT1
Chen et al. 2011 [108]	BALB/c mice; PC-3 cells; tail vein injection; histopathological and X-ray analysis	OLFM4 expression leads to a reduced bone metastasis via an inhibition of CXCL12-mdiated AKT phosphorylation
Chen et al. 2013 [112]	CB17-SCID mice; PC-3 cells; intratibial injection; X-ray analysis and histological analysis	Cells with a high CCN3 expression exhibit an increased bone metastasis and osteoclast formation via a RANKL-mediated activation of the FAK/AKT/ p38/NF-κB axis in osteoclasts
Chen et al. 2012 [113]	CB17-SCID mice; PC-3 cells; intratibial injection; X-ray analysis	Knockdown of CCN3 decreases the formation of osteolytic lesions via an absent CCN3-mediated AKT activation
Xu et al. 2018 [116]	BALC/c mice; DU145 cells; subcutaneous injection; ELISA analysis of serum and IHC analyasis	Inhibition of AKT reduces expression of BSP, OPN, MMP2 and αvβ3-integrin
Colden et al. 2017 [119]	Athymic nude mice; PC-3 cells; orthotopic or intracardiac injection; BLI	miR-466 suppresses bone metastasis via a downregulation of RUNX2 and RUNX2-mediated AKT expression
Josson et al. 2014 [120]	Nude mice; ARCaPM cells; orthotopic injection, BLI and IHC analysis	miR-409-3p/-5p expression upregulates pAKT levels and inhibition of miR-409-3p/-5p leads to a decreased bone metastasis
Jin et al. 2015 [123]	Nude mice; PC3-MM2 cells; intracardiac injection; BLI and X-ray analysis	Inhibition of S435 phosphorylation of Talin reduced bone metastasis accompanied by lower pAKT levels
Mainetti et al. 2015 [124]	Injection into human fetal femur fragments implanted into SCID mice; PC-3, and C4-2B cells; histological analysis	Expression of c-kit increases bone metastasis partly via the PI3K/AKT pathway
Seol et al. 2019 [129]	Athymic nude mice; PC-3 cells; intracardiac injection; PCR analysis of hindlimb DNA	Overexpression of the IL-7R increases bone metastasis partly via an activation of the AKT signaling
Liao et al. 2006 [130]	Athymic nude mice; PC-3 cells; intracardiac inoculation; BLI	Knockdown of CaSR reduces bone metastasis via a reduced calcium-dependent AKT activation
Tang et al. 2018 [131]	BALB/c mice; PC-3 cells; intracardiac injection; BLI and X-ray analysis	miR-133a-3p reduces the formation of osteolytic lesions via a suppression of EGFR- and IGF-1R-mediated AKT activation
Pradhan et al. 2018 [134]	Athymic nude mice; PC3-ML cells; intracardiac injection; BLI	Overexpression of AKT increases bone metastasis and treatment with MDA-7/IL-24 abrogates the increased bone metastasis
Liu et al. 2019 [136]	BALB/c mice; DU145 cells; intratibial injection; micro-CT and histological analysis	The PI3K inhibitor ZSTK474 suppresses bone metastasis and osteolysis via an inhibition of the AKT-mediated MMP9 expression
Mancini et al. 2018 [100]	CD1 nude mice; PC-3 or 22v1 cells; intracardiac or intratibial injection; X-ray analysis	The dual PI3K/mTOR inhibitor X480 inhibits bone metastasis and tumor-induced osteolysis
Watanabe et al. 2016 [137]	BALB/c mice; PC-3 cells; intratibial injection, micro-CT, DEXA and histological analysis	The tyrosine kinase inhibitor TAS-115 reduced cancer-induced osteolysis via an inhibition of the M-CSF/FMS-dependent AKT activation in osteoclasts
Huang et al. 2015 [138]	SCID mice; PC-3 cells; intratibial injection; X-ray and histological analysis	Pristimerin inhibits the formation of osteolytic bone metastases via a reduction of VEGFR-mediated AKT signaling in bone marrow endothelial progenitor cells
Hsu et al. 2015 [139]	SCID mice; PC-3 cells; intratibial injection; micro-CT and X-ray analysis	Inhibition of IL-20 mediated AKT activation reduces cancer-induced osteolysis
Yano et al. 2008 [141]	Nude mice; PC-3M cells; intratibial injection; BLI	The Hsp90 inhibitor 17AAG increases the intraosseous tumor growth via a Src-dependent AKT activation in osteoclasts
Dayyani et al. 2012 [143]	Nude mice; PC3-MM2 cells; intratibial injection; micro-CT and X-ray analysis and analysis of bone turnover parameters	Combined inhibition of IGF-1R and Src family kinases inhibits the formation of osteolytic lesions and bone turnover via a suppression of AKT1 and AKT2 activity

	Rabbani et al. 2010 [144]	SCID mice; PC-3 cells; intratibial inoculation; micro-CT and X-ray analysis	The Src/Abl kinase inhibitor bosutinib inhibits the formation of osteolytic lesions via reduced pAKT levels and reduced expression of MMP9, uPAR and IL-8
	Rabbani et al. 2010 [145]	SCID mice; PC-3 cells; intratibial inoculation; X-ray and histological analysis	The uPAR inhibitor ATN-658 reduces the intraosseous tumor growth partly via an inhibition of AKT phosphorylation
	Yates et al. 2015 [146]	Athymic mice; PC3-ML; intracardiac injection; BLI	4-methylumbelliferone inhibits bone metastasis partly via a suppression of the AKT pathway
	Hsieh et al. 2020 [147]	BALB/c mice; PC-3 cells; intratibial injection; BLI and histological analysis	LCC03 inhibits the intraosseous tumor growth via an enhanced autophagy through a reduced AKT signaling
	Banerjee et al. 2007 [148]	Injection into human fetal femur fragments implanted into SCID mice; C4-2B cells; tumor volume and histological analysis	Combination of docetaxel and the ETA inhibitor ABT-627 reduces intraosseous tumor growth via a suppression of the AKT/NFKB axis
lung cancer	Nakamura et al. 2006 [153]	SCID mice; SBC-5 cells; tail vein injection; histological analysis	CCR4 expressing tumor cells colocalizes with CCL22 producing osteoclasts (CCL22 activates the AKT signaling via CCR4)
	Chang et al. 2017 [159]	SCID mice; A549 cells; orthotopic injection; BLI	Querceptin reduces bone metastasis via a downregulation of Snail followed by a maspin-mediated inhibition of AKT activity

Table S2. Clinical experiments evaluating the role of AKT in bone metastasis of breast, prostate and lung cancer. Information in brackets represent data of in vitro or in vivo experiments of the respective publication.

Type of solid tumor	Publication	Study design	Main findings
breast cancer	Chen et al. 2018 [51]	Microarray gene expression analysis of disseminated tumor cells and bone metastatic tumor cells	The PI3K/AKT pathway is associated with the formation of bone metastases
	Kono et al. 2018 [52]	Next generation sequencing of patients with bone metastases, non-bone metastases or bone and non-bone metastases	The frequency of somatic mutations of AKT and PIK3CA show no differences between bone metastases and non-bone metastases
	Li et al. 2020 [53]	Microarray analysis of primary tumor and different sites of metastasis	AKT signaling is downregulated in bone metastases and upregulated in skin metastases
	Wang et al. 2015 [54]	Expression analysis of various metastatic sites and The Cancer Genome Atlas analysis for signature scores in disseminated tumor cells and bone metastases	N cadherin levels are higher in bone metastases compared to other metastatic sites and high E cadherin levels are associated with a shorter bone metastasis-free survival (E-N-heterotypic adherens junctions promote bone metastasis via the activation of the AKT/mTOR signaling) mTOR activity is higher in bone metastases compared to disseminated tumor cells
	Werner et al. 2015 [55]	Gene expression profiling primary tumor and disseminated tumor cells in the bone marrow	Downregulation of RAI2 is more frequent in disseminated tumor cells in the bone marrow compared to primary tumor probes (Knockdown of RAI2 increases pAKT levels)
	Sun et al. 2019 [56]	Database analysis of primary tumor and bone metastases	Bone metastases show higher CENPF expression compared to primary tumor or lung metastases (CENPF promotes PTHrP expression via PI3K/AKT/mTORC1 activation)
	Kim et al. 2006 [58]	IHC staining of primary breast tumors with simultaneous or non-simultaneous bone metastases	pAKT levels are elevated in 81.8% of HER2-positive breast cancer with bone metastases (The HER2/CXCR4/AKT axis is suggested to be important in bone metastases)
	Cabioglu et al. 2005 [61]	Expression analysis of breast tumor patients	A subpopulation of patients with breast cancer exhibits a co-expression of CXCR4 and HER2 (The CXCL12/CXCR4 partly activates the AKT signaling)
	Zhang et al. 2009 [63]	Gene expression analysis of a dataset of breast tumors with known bone metastasis outcomes. Microarray gene expression analysis of bone, brain and lung metastases	A Src response signature is associated with a bone relapse and the formation of bone metastases in patients. Expression of CXCL12, IGF1, BMP2, TGFβ, PDGF, VEGF and TRAIL is higher in bone metastases compared to other distant metastatic sites (Knockdown of c-Src reduces bone metastasis partly via a diminished CXCL12-dependent AKT activation)
	Zhang et al. 2013 [64]	Gene expression analysis of various datasets of breast tumors and The Cancer Genome Atlas analysis	CXCL12 and IGF1 are associated with a lower bone metastasis-free survival and with a Src response signature. CXCL12 and IGF1 originates from cancer associated fibroblasts (CAF) and a CAF signature is overrepresented in bone metastases compared to other metastatic sites as well as is associated with a lower bone metastasis-free survival (Knockdown of c-Src reduces bone metastasis partly via a diminished CXCL12-dependent AKT activation)
	Huang et al. 2017 [80]	IHC analysis of breast tumor samples	Expression of BMP2 is positively correlated with the expression of CD44 and is negatively correlated with the expression of Rb (BMP2 promotes the degradation of Rb via the AKT pathway and therefore causes an upregulation of EMT-promoting CD44)
	Yamaguchi et al. 2016 [83]	Expression analysis of breast cancer patients	A low regucalcin expression is associated with a poor prognosis (Regucalcin overexpression reduced AKT signaling and suppressed osteoclastogenesis)

	Humphries et al. 2020 [84]	Gene expression analysis of CTCs derived from metastatic breast cancer patients and The Cancer Genome Atlas analysis	High expression of genes, which are known to drive mitochondrial fission, correlate with a longer metastasis-free survival (Mitochondrial fission decreases the formation of osteolytic bone metastases via a reduced AKT activation)
	Feng et al. 2016 [90]	The Cancer Genome Atlas analysis and gene set enrichment analysis	A high expression of FAF1 is associated with a better metastasis-free survival and a lower tumor invasiveness. A consensus signature of genes correlated with bone metastasis is enriched in FAF1-low patients
prostate cancer	Mancini et al. 2018 [100]	IHC staining of pAKT in bone metastases vs. primary tumor	Higher activation of AKT in bone metastases compared to the primary tumor
	Mimeault et al. 2012 [103]	IHC and immunofluorescence staining in normal prostate tissue, primary tumor and bone metastases	Higher pAKT levels in bone metastases compared to normal prostate tissue and primary tumor
	Ihle et al. 2019 [104]	Immunofluorescence staining of lytic vs. blastic lesions of prostate cancer	Osteolytic lesions exhibit higher pAKT levels and osteoblastic lesions show higher pSTAT3 levels
	Chen et al. 2013 [112]	IHC staining in normal tissue, early-stage prostate cancer, late-stage prostate cancer and bone metastases	CCN3 levels are higher in bone metastases compared to normal tissue, early stage and late stage prostate cancer (CCN3 increases osteoclastogenesis via the RANKL-dependent AKT activation)
	Zhang et al. 2011 [117]	IHC staining in primary tumor, lymph node metastases and bone metastases	Specimen of lymph node metastases and bone metastases exhibit lower PTEN levels (therefore a higher AKT activation) and higher RUNX2 levels compared to the primary tumors
	Yamaguchi et al. 2021 [122]	Microarray data of primary tumor and metastases	Lower regucalcin expression in the metastases compared to the primary tumor. (Overexpression of regucalcin reduces AKT-mediated bone cell stimulation)
	Jin et al. 2015 [123]	IHC staining of primary tumor, lymph node metastases and bone metastases	Higher pTalin S425 levels in bone metastases compared to primary tumor and lymph node metastases (Talin phosphorylation is associated with AKT phosphorylation)
	Mainetti et al. 2015 [124]	RT-PCR analysis of bone metastases and surrounding stroma	Expression of c-kit is enhanced in bone metastases (c-lit activates the PI3K/AKT pathway)
	Dolloff et al. 2007 [125]	Prostate cancer cells were cultured with bone marrow from a healthy male	Bone-metastatic prostate cancer cells exhibit higher pAKT levels after stimulation with human bone marrow via α-PDGFR activation
	Seol et al. 2019 [129]	Expression data of The Cancer Genome Atlas	Expression of IL-7 and IL-7Rα (and therefore AKT activation) is associated with cancer stem cells and EMT
	Tang et al. 2018 [131]	Expression data of The Cancer Genome Atlas and analysis of bone metastasis-free survival of patients with low or high miR-133a-3p expression	miR-133a-3p levels are lower in patients with bone metastases compared to patients without bone metastases Low levels of miR-133a-3p are associated with a lower bone metastasis-free survival (miR-133a-3p expression suppresses AKT activation)
lung cancer	Liu et al. 2020 [152]	Gene chip and quantitative PCR analysis of miR-365 serum levels in patients with bone metastases	Serum miR-365 levels are reduced in patients with bone metastases (Downregulation of mir-365 promotes the EGFR/PI3K/AKT through NKX2-1)
	Choi et al. 2020 [154]	Next generation sequencing, Sanger sequencing and quantitative real-time PCR analysis of bone metastases	RANKL expression is higher in bone metastases with a GNAQ mutant compared to bone metastases with GANQ wildtype
	Chang et al. 2017 [159]	Expression data of The Cancer Genome Atlas	High levels of Snail and pAKT in patients with lung cancer are associated with a shorter survival time
